# De novo genome assembly of the invasive mosquito species *Aedes japonicus* and *Aedes koreicus*

**DOI:** 10.1186/s13071-023-06048-w

**Published:** 2023-11-20

**Authors:** Paolo L. Catapano, Monica Falcinelli, Claudia Damiani, Alessia Cappelli, Despoina Koukouli, Paolo Rossi, Irene Ricci, Valerio Napolioni, Guido Favia

**Affiliations:** 1 School of Biosciences and Veterinary Medicine, University of Camerino, Via Gentile III da Varano, 62032 Camerino, Italy; 2 School of Biosciences and Veterinary Medicine, University of Camerino, CIRM Italian Malaria Network, Via Gentile III da Varano, 62032 Camerino, Italy

**Keywords:** Aedine, Assembly, Genome, Insecticide resistance, Thermal stress

## Abstract

**Background:**

Recently, two invasive *Aedes* mosquito species, *Ae. japonicus* and *Ae. koreicus*, are circulating in several European countries posing potential health risks to humans and animals. Vector control is the main option to prevent mosquito-borne diseases, and an accurate genome sequence of these mosquitoes is essential to better understand their biology and to develop effective control strategies.

**Methods:**

A de novo genome assembly of *Ae. japonicus* (Ajap1) and *Ae. koreicus* (Akor1) has been produced based on a hybrid approach that combines Oxford Nanopore long-read and Illumina short-read data. Their quality was ascertained using various metrics. Masking of repetitive elements, gene prediction and functional annotation was performed.

**Results:**

Sequence analysis revealed a very high presence of repetitive DNA and, among others, thermal adaptation genes and insecticide-resistance genes. Through the RNA-seq analysis of larvae and adults of *Ae. koreicus* and *Ae. japonicus* exposed to different temperatures, we also identified genes showing a differential temperature-dependent activation.

**Conclusions:**

The assembly of Akor1 and Ajap1 genomes constitutes the first updated collective knowledge of the genomes of both mosquito species, providing the possibility of understanding key mechanisms of their biology such as the ability to adapt to harsh climates and to develop insecticide-resistance mechanisms.

**Graphical Abstract:**

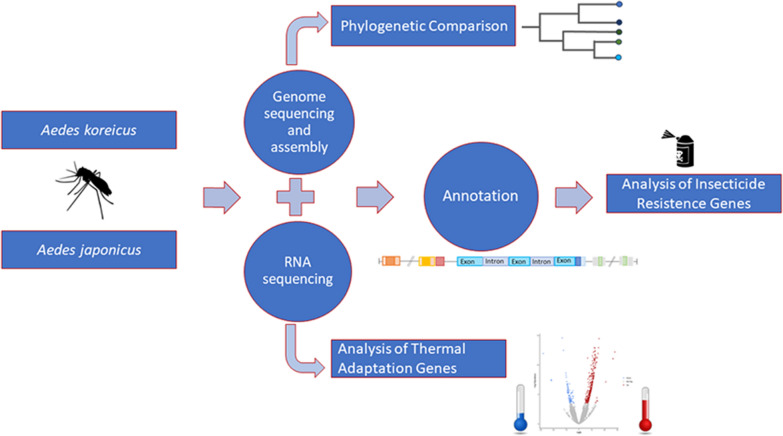

**Supplementary Information:**

The online version contains supplementary material available at 10.1186/s13071-023-06048-w.

## Background

In the early 1990s, Europe experienced the colonization of vast continental areas by the *Aedes albopictus* mosquito [1]. This mosquito of non-European origin is now permanently resident in the continent, and it has been the protagonist of some viral epidemics as vector of numerous arboviruses, such as chikungunya, zika, dengue and also of heartworms diseases, such as filariosis [1]. Also, the sudden spread in Africa of *Anopheles stephensi*, a major Asian malaria vector, is generating outbreaks even in those areas where malaria was almost eradicated [2, 3]. Being adapted to urban life, *An. stephensi* has the potential to further spread to many urban areas across the African continent, implying an increased number of people at risk of malaria [4]. Conceivably, climate change may influence the adaptation of other invasive species to new environmental niches, thus colonizing new areas and rapidly spreading to others. In this frame, the appearance in Europe of two *Aedes* invasive species, namely *Ae. japonicus* and *Ae. koreicus*, is not surprising. The European advent of *Ae. japonicus* is dated to 2000, in France [5], while *Ae. koreicus* was reported in Belgium in 2008 [6]. Since then, established populations of *Ae. japonicus* were detected in Belgium, Germany, Switzerland, Austria, Slovenia, Croatia, The Netherlands, Italy, Hungary, Luxembourg and Northern Spain. *Aedes koreicus* is well established in Italy, Germany, Russia and Hungary, and it has also been found in Slovenia and Switzerland [7]. Moreover, *Ae. koreicus* has been passively spreading along the European route E35 from Italy to Germany while *Ae. japonicus* has been expanding through active dispersal [8]. Thus, both species show a remarkable ability to adapt to different eco-environmental and climate conditions. Nevertheless, despite their potential role in the transmission of endemic and imported pathogens, not much is known about their real vectorial capacity and their ability to adapt to specific environmental conditions and eco-ethological contexts.

To understand the basic biology of these species, more research is needed, given the possible development of control strategies. Although a first draft of the genome of *Ae. koreicus* has been already published [9], the above-reported scenario prompted us to conduct a study aimed at sequencing the genome of these two invasive species, with a special focus to gene clusters of peculiar interest such as those responsible for insecticide resistance and thermal adaptation.

## Methods

### Collection of samples

Larvae of *Ae. koreicus* and *Ae. japonicus* were collected in two villages situated in Veneto region (northeast Italy), Alano di Piave (45° 54′ 26″ N, 11° 54′ 28″ E) and Feltre (46° 0′ 49.903″ N 11° 53′ 49.996″ E), respectively. We performed our studies on mosquitoes collected as larvae from the same pond and then reared in our insectarium until the adult stage. This heavily reduced the heterogeneity among samples. Fourth-instar larvae were morphologically identified according to Montarsi et al. [10]. The DNA of newly emerged adults was used for the genome analysis. The RNA of fourth-instar larvae and adults was used for the RNA sequencing analysis.

Total genomic DNA was obtained from pools of three females of *Ae. koreicus* and *Ae. japonicus*. DNA was extracted using a JetFlex Genomic DNA Purification kit (Invitrogen, Thermo Fisher Scientific, Waltham, MA, USA) according to the manufacturer’s instructions.

For the RNA-seq analysis, samples were prepared from two different cohorts of adults and fourth-instar larvae *Ae. koreicus* and *Ae. japonicus*. Adults were reared at 15 °C and 28 °C, respectively, for 5 days. Larvae were incubated for 8 h at 4 °C and 28 °C, respectively. Total RNAs were extracted from a single adult and pool of five fourth-instar larvae using RNAzol reagent (Sigma-Aldrich USA), according to the manufacturer’s instructions.

### Sequencing

Samples of *Ae. koreicus* and *Ae. japonicus* were sent to IGAtech (Udine, Italy) for short- and long-read sequencing. The same samples were used for both analyses. Briefly, for short-read sequencing, CeleroTM DNA-Seq kit (NuGEN, San Carlos, CA) has been used for library preparation following the manufacturer’s instructions and was sequenced with NovaSeq 6000 in paired-end 150 bp mode. Long-read sequencing was performed with Oxford Nanopore PromethION. The samples were prepared using the SQK-LSK109 kit and sequenced on a flowcell R9.4.1. Basecalling of data was performed with Guppy 5.0.13. Reads were filtered with minimum qscore of 7 and length of 500.

For RNA-seq sequencing, Universal Plus mRNA-Seq kit (Tecan Genomics, Redwood City, CA) has been used for library preparation. Libraries were then sequenced with a paired-end 150-bp mode on NovaSeq 6000 (Illumina, San Diego, CA).

The total reads count generated by NovaSeq 6000, and Oxford Nanopore PromethION, N50 of reads, and total Mbps sequenced are reported in (Additional file 1: Table S1A–C).

### Assembly

The Illumina reads were error-corrected using BFC release 181, adjusting the parameters based on the total draft genome size [11]. The Nanopore reads were error-corrected using LoRDEC v0.9 [12] with the error-corrected Illumina overlapping Paired-End (PE) reads, a k-mer size of 19 and a solidity threshold of 3. A first assembly was performed using FLYE 2.9.1 [13] with the long raw Oxford Nanopore reads. The resulting assembly was polished using two rounds of HyPo v1.0.3 [14] with the error-corrected Illumina overlapping PE reads. Scaffolding was then performed with two rounds of LINKS v2.0.1 [15] and ntLINKS v1.3.4 [16] using the gap-filling option with the error-corrected Illumina PE and Nanopore libraries. An additional round of HyPo, as previously described, was performed. We removed haplotig contamination by using “purged" dups [17] to produce the final assembly. The assembly quality was assessed by computing two metrics: QUAST v5.0.2 [18] and BUSCO v4.0.5 [19], using as reference lineage Diptera.

### RNA-seq analysis

Adapter sequences and low-quality bases were trimmed (Trimmomatic) [20] and quality checks before and after trimming were performed (Fastqc) [21]. De novo assemblies were done with SPAdes [22] for RNA. Quality of assemblies was assessed with BUSCO [19] and QUAST [18]. Error-corrected reads were mapped to transcripts, and read count was performed with SALMON [23]. Read count files were then used to compare gene expression of two groups, cold (15 °C) and hot (28 °C), using DESeq2 pipeline in RStudio [24]. We performed transcript annotation using Trinotate pipeline [25]. Gene ontology and functional annotation were done with UniProt [26] and Enrichr [27].

### Annotation

The automatic annotation of the genome was performed using the MAKER pipeline [28]. We provided MAKER data with RNA-seq data of *Ae. koreicus* and *Ae. japonicus* sequenced by our laboratory and additional ESTs and RNA-seq data from similar mosquito species such as *Aedes aegypti*, *Ae. albopictus* and *Culex quinquefasciatus* that were publicly available. Coherently, we have performed a de novo transcriptomic assembly to integrate and improve the genome annotation quality.

To predict gene models, SNAP [29] and Augustus [30] within MAKER were used. To detect repetitive elements in the genomes, we used RepeatMasker, version 4.1.4, using the open source library Dfam [31]. Functional annotation was performed with InterProScan 5 [32].

### Phylogenetic analysis

Comparative genomic analysis was performed with the Orthofinder pipeline, consisting of a combination of algorithms called STAG and STRIDE, to infer a species rooted phylogenetic tree [33]. The two new assemblies of *Ae. koreicus* and *Ae. japonicus* were compared with mosquito genomes previously assembled like *Ae. aegypti*, *Ae. albopictus*, *Cx. quinquefasciatus*, *Anopheles gambiae*, *An. coluzzii*, *An. arabiensis* and *An. darlingi* and using *Drosophila melanogaster* genome as an outgroup. The phylogenetic tree graph was generated using iTOL v6 [34].

### Detection of cold tolerance genes and insecticide resistance genes in the new assemblies

For the detection of the cold tolerance genes, protein sequences of genes found to be differentially expressed in *D. melanogaster* at cold temperatures compared to warmer temperatures [35] were mapped [36] to the assemblies of *Aedes koreicus*, *Ae. japonicus*, *Ae. albopictus* and *Ae. aegypti* using TBLASTN, on both plus and minus strands to improve alignment of sequences between different species. To remove false hits, we filtered results based on hit scoring (> 200), and in the case of multiple hits in the same genomic location, only the hit with the highest score was retained. To find the genes specific to the different mosquito species, we compared the results and built Venn diagrams with RStudio. Functional annotation enrichment analysis was performed with Flyenrichr [27].

For the detection of insecticide resistance genes in the new assemblies, we mapped the protein sequences of 751 metabolic insecticide resistance genes of *Ae. aegypti* [37] using TBLASTN [34] against the assemblies of *Ae. koreicus*, *Ae. japonicus* and *Ae. albopictus*. We filtered results based on hit scoring (> 200), and if two hits occurred in the same genomic location, we kept the hit with the highest score. We then compared results between species to detect genes specific to *Ae. koreicus* and *Ae. japonicus* and not present in *Ae. albopictus*. Venn diagrams were built with RStudio.

## Results

### Genome length and GC content

Using a hybrid approach that combines Oxford Nanopore long reads and Illumina short reads data, we assembled a scaffold-level version of *Ae. koreicus* and *Ae. japonicus* genomes whose size was assessed as 1.24 and 1.39 gigabase (Gb) pairs, respectively. These dimensions resemble those of other aedines such as *Ae. aegypti* and *Ae. albopictus*, which are estimated to be respectively 1.22 [38] and 1.19–1.28 Gb pairs [19, 39]. The GC content of the two genomes is very similar: 39.68% in *Ae. koreicus* while 39.51% in *Ae. japonicus*. Again, these metrics are comparable to those of *Ae. aegypti* (38.3%) and *Ae. albopictus* (40.4%). Genome completeness of the two species, measured using Benchmarking Universal Single-Copy Orthologs (BUSCO) [19], showed a gene completeness for *Ae. koreicus* of 91.8%, and 8% of duplicates, while for *Ae. japonicus*, it showed 92.5% gene completeness and 13.6% duplicates. Genome annotation yielded 18,647 and 18,687 genes for *Ae. koreicus* and *Ae. japonicus*, respectively. The N50 values are 190,716 for *Ae. koreicus* and 118,241 for *Ae. japonicus* and coverage is 35X and 20X, respectively (Table 1). By using RepeatMasker in *Ae. japonicus* and *Ae. koreicus*, we detected 71% and 71.92% of the genomes, respectively, as repetitive DNA. These data are similar to what was reported in *Ae. albopictus*, (74%) [39], but much higher than reported in *Ae. aegypti* (64%) [38] and *Culex tarsalis* (60,8%) [40].

**Table 1:** Main genome features of *Aedes koreicus* and *Ae. japonicus*. (A) Metrics assembly and (B) BUSCO Score of *Ae. koreicus* and *Ae. japonicus*, respectively

A		
Metrics	*Aedes koreicus*	*Aedes japonicus*
Assembly size	1.24 Gbp	1.39 Gbp
N50^a^	190,716 bp	118,241 bp
Number of scaffolds	21,315	25,255
GC% content	39.68%	39.51%
Repetitive elements^b^	71.92%	71.00%
Genes predicted^c^	18,647	18,687
Coverage	35X	20X

B		
BUSCO metric	Number of genes	Percentage
BUSCO assessment results of *Aedes koreicus*		
Completeness	3016	91%
Single copy	2753	84%
Duplicate	263	8%
Fragmentated	74	2%
Missing	195	6%
Number of genes in BUSCO database diptera	3285	
BUSCO assessment results of *Aedes japonicus*		
Completeness	3038	92,5%
Single copy	2591	79%
Duplicate	447	13,6%
Fragmentated	79	2%
Missing	168	5%
Number of genes in BUSCO database diptera	3285	

^a^Determined by QUAST

^b^Determined by RepeatMasker

^c^Determined by MAKER

### Phylogenetic analysis

**Fig. 1:**
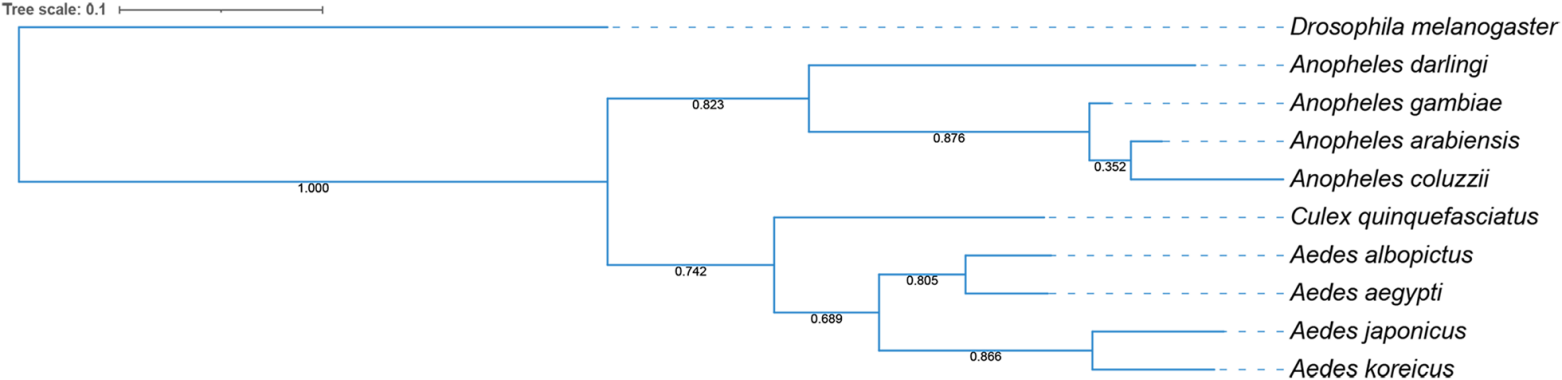
Phylogenetic analysis of *Aedes koreicus* and *Ae. japonicus* genomes comparing to known mosquito species. This phylogenetic analysis consists of an Orthofinder Stag and Stride algorithm comparing *Ae. koreicus* and *Ae. japonicus* with other genome-sequenced mosquito species. *Drosophila melanogaster* was used as outgroup. Bootstrap values are shown at each branch

The phylogenetic analysis was based on the comparison between the predicted proteomes of *Ae. koreicus* and *Ae. japonicus* and the proteomes of seven mosquito species belonging to three different genera (*Anopheles gambiae*, *An. coluzzii*, *An. arabiensis*, *An. darlingi*, *Aedes aegypti*, *Ae. albopictus* and *Culex quinquefasciatus*). As expected, phylogenetic clustering revealed two main clades. Clade I contains the four *Anopheles* species while Clade II contains the four *Aedes* species and *Cx. quinquefasciatus*. Notably, the phylogenetic tree showed that the new invasive species, *Ae. koreicus* and *Ae. japonicus*, share a most common recent ancestor (MCRA). Notably, the MCRA of *Ae. koreicus* and *Ae. japonicus* diverged more recently compared to the MCRA of *Ae. aegypti* and *Ae. albopictus* (Fig. 1).

### Annotation of specific genes

Among the several different classes of genes, particular attention has been devoted to two specific classes of genes: the ones involved in thermal adaptation and those involved in insecticide resistance.

#### Thermal adaptation genes

**Fig. 2:**
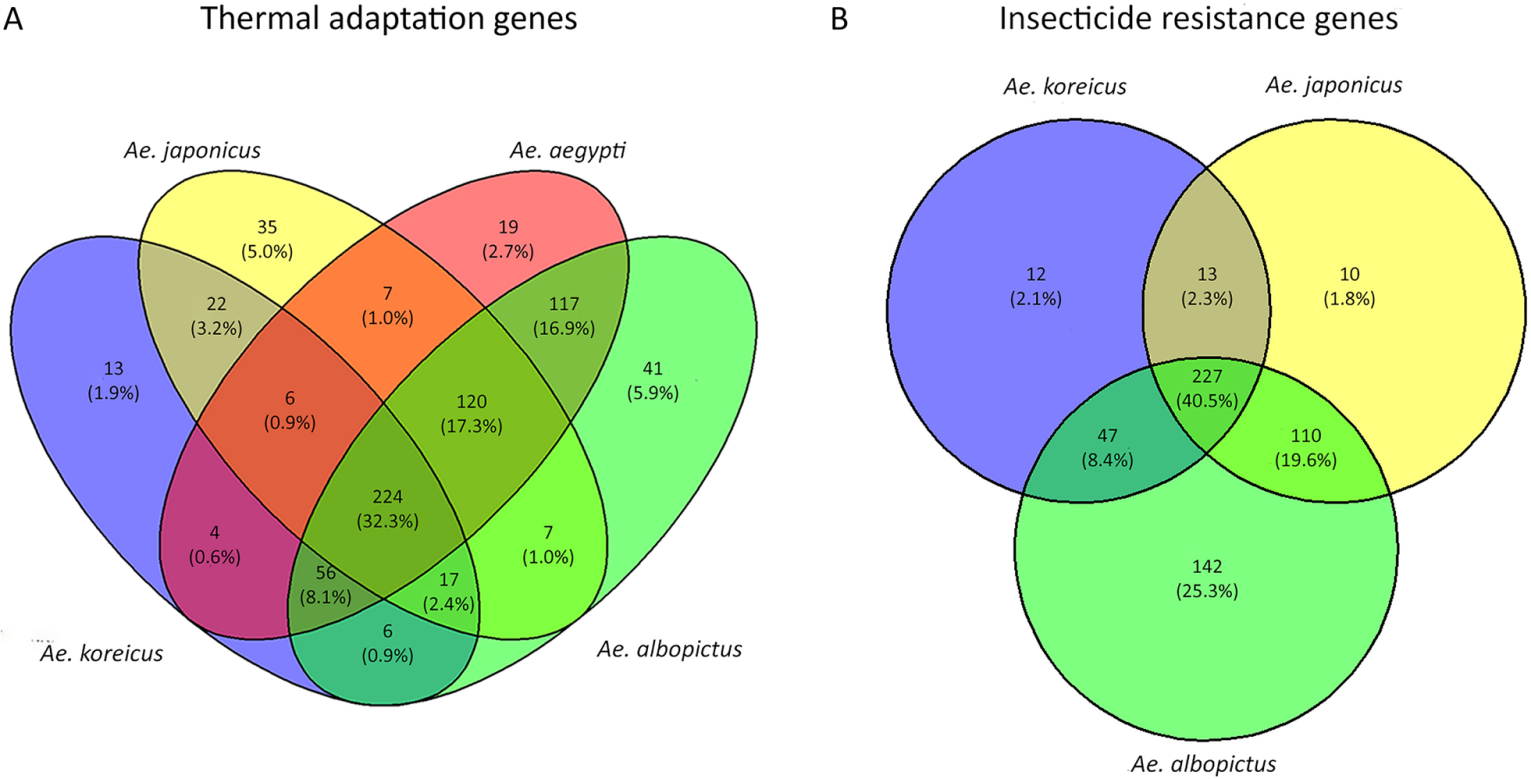
Convergence analysis of genes involved in **A** thermal adaptation and **B** insecticide resistance. Venn diagrams show species-specific (unique) or shared genes among *Aedes koreicus*, *Ae. japonicus*, *Ae. aegypti* and *Ae. albopictus* mosquitoes. Thermal adaptation genes refer to *Drosophila melanogaster* and detected by homology-based search in the mosquito genomes. Insecticide resistance genes refer to *Ae. aegypti* and detected with BLAST in the genomes of *Ae. koreicus*, *Ae. japonicus* and *Ae. albopictus*

The comparative analysis of genes involved in thermal stress between the two invasive species was extended also to *Ae. albopictus* and *Ae. aegypti* using *Drosophila melanogaster* as reference organism [35]. Indeed, of 694 selected genes in *Drosophila*, we have identified 348 homologous genes in *Ae. koreicus* (Additional file 2: Table S2A) and 438 genes in *Ae. japonicus* (Additional file 2: Table S2B). Of these, 13 are specific to *Ae. koreicus* (Additional file 2: Table S2C) and 35 to *Ae. japonicus* (Additional file 2: Table S2D). An additional 22 genes are shared between these two species but not with *Ae. aegypti* and *Ae. albopictus* (Additional file 2: Table S2E) (Fig. 2A). Particularly, the 13 specific genes of *Ae. koreicus* are enriched in KEGG pathways involved in pentose phosphate pathway, fructose, mannose, galactose metabolism, neuroactive ligand-receptor interaction, RNA degradation and glycolysis/gluconeogenesis (Additional file 3: Table S3A). The 35 specific genes of *Ae. japonicus* are enriched in KEGG pathways such as glycerophospholipid metabolism and aminoacyl-tRNA biosynthesis (Additional file 3: Table S3B). The 22 genes shared between these two species were enriched for KEGG pathways involved in tryptophan, phenylalanine, tyrosine and pyruvate metabolism (Additional file 3: Table S3C).

#### Insecticide resistance genes

The comparative analysis of genes involved in insecticide resistance between the two invasive species extended also to *Ae. albopictus* using *Ae. aegypti* as reference organism [37]. Of 561 genes, we have identified 299 genes in *Ae. koreicus* (Additional file 4: Table S4A) and 360 genes in *Ae. japonicus* (Additional file 4: Table S4B). Of these, 12 are specific to *Ae. koreicus* and 10 to *Ae. japonicus* (Additional file 4: Table S4C and D respectively). An additional 13 are shared between these two species but not with *Ae. albopictus* (Additional file 4: Table S4E) (Fig. 2B). All these genes belong to gene families known to be involved in lower insecticide penetration, sequestration and biodegradation: genes encoding for detoxification enzymes (P450, CCEs, GSTs, UGTs), cuticle proteins, ATP-binding cassette (ABC) transporters, neurotransmitter receptors and voltage-gated channels. All three species share most of these families; nevertheless, the differential distribution of members of the individual families characterizes the relative composition and quantities. In more detail, 4 of 12 of the specific genes of *Ae. koreicus* belong to “cuticle gene family” (chitin synthase, pupal cuticle protein putative, pupal cuticle protein E78 putative, puticle protein putative), 3 to “ion channel gene family” (sodium leak channel non-selective protein, voltage-dependent L-type calcium channel subunit alpha, voltage-gated potassium channel) and 2 to “ABC transporter gene family” (ATP-binding cassette sub-family A member 3, multidrug resistance protein 2/ATP-binding cassette protein c) while 2 were classified as “other detox genes” (short-chain dehydrogenase) and one more as “other gene” (modifier of *mdg4*).

In *Ae. japonicus* we specifically identified one member of the “ABC transporter family” (multidrug resistance protein 2/ATP-binding cassette protein c), one member of the “cuticle family” (pupal cuticle protein, putative), two are ion channels (voltage-gated potassium channel, glutamate-gated chloride channel) and two P450 gene (cytochrome P450), and of the last four remaining genes, three were categorized as “other detox” [sterol desaturase, NAD(P)H oxidase (H(2)O(2)-forming, Oxidoreductase] and one as “other” (BTB domain-containing protein). Thirteen genes are specifically shared only by *Ae. koreicus* and *Ae. japonicus*; one is an ABC transporter (ABC transporter), three belong to cuticle family (brain chitinase and chia, chitin synthase, pupal cuticle protein putative), two are ion channels (voltage-dependent L-type calcium channel subunit alpha) and one is a P450 gene (cytochrome P450). Finally, 3 of 13 shared genes are synaptic receptors (nicotinic acetylcholine receptor, putative, nicotinic acetylcholine receptor beta-2 subunit putative), two are oxidoreductase (heme peroxidase, thioredoxin reductase) and one belongs to the “other detox” family (sterol desaturase).

### RNA-seq analysis of thermal adaptation genes

**Fig. 3:**
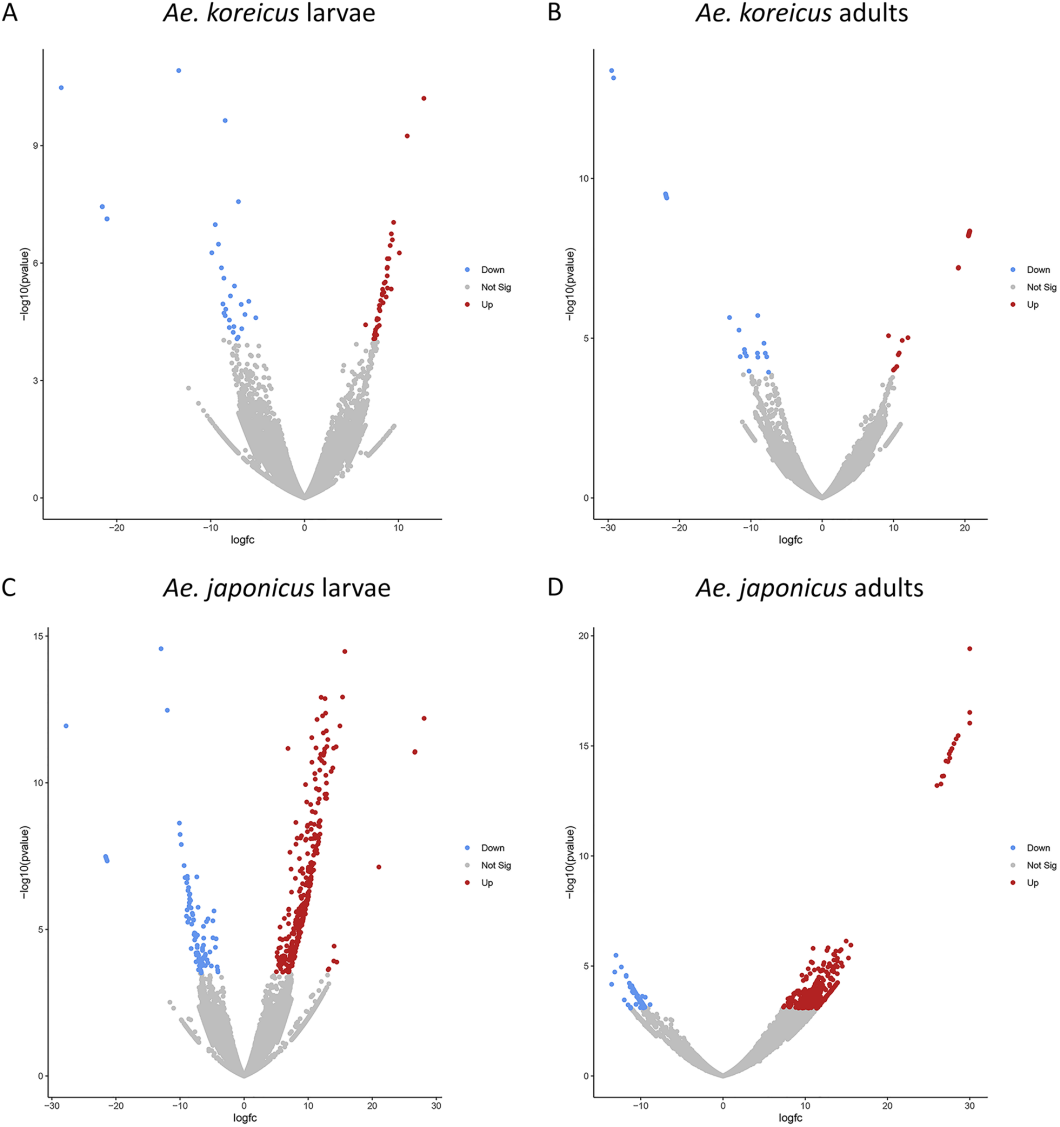
Differentially expressed thermal adaptation genes in larvae and adults of *Aedes koreicus* and *Ae. japonicus*. Volcano plots comparing transcripts obtained by samples reared at 4 °C (larvae) and 15 °C (adults) and 28 °C (larvae and adults control groups): upregulated (red), downregulated (blue) and not significantly altered (gray) transcripts are shown in **A** *Ae. koreicus* larvae, **B** *Ae. koreicus* adults, **C** *Ae. japonicus* larvae and **D** *Ae. japonicus* adults, respectively

Through the RNA-seq analysis of larvae and adults of *Ae. koreicus* and *Ae. japonicus* exposed to different temperatures (15 and 28 °C), we identified genes showing a differential temperature-dependent activation (Fig. 3). In detail, under the low-temperature stress of 15 °C, several genes were differentially expressed and the relative encoded proteins identified: in *Ae. koreicus* larvae (Additional file 5: Table S5A), of 57 upregulated and 40 downregulated transcripts, we identified 8 upregulated and 4 downregulated proteins. In adults, among 225 upregulated and 26 downregulated transcripts, we identified 27 upregulated and 2 downregulated proteins (Additional file 5: Table S5B). Larvae and adults share only one upregulated gene (*aef1*, adult enhancer factor 1) but none of the downregulated (Table 2). In *Ae. japonicus* larvae, out of 325 upregulated and 101 downregulated transcripts, we identified 373 upregulated and 14 downregulated proteins (Additional file 5: Table S5C). In adults, among 502 upregulated and 79 downregulated transcripts, we identified 70 upregulated and 24 downregulated proteins (Additional file 5: Table S5D). Larvae and adults share 18 upregulated genes and 3 of the downregulated (Table 2). Functional GO terms were enriched in eight molecular functions and one cellular component in larvae of *Ae. koreicus* while two molecular functions in adults. Instead, in *Ae. japonicus* larvae, three biological processes and one molecular function are enriched, while no enrichment was present in adults of *Ae. japonicus* (Additional file 6: Table S6). Notably, several differentially expressed genes seem strongly involved in thermal adaptation as they plausibly encode for specific proteins: in larvae of *Ae. koreicus*, among others of interest, we found upregulated genes encoding for alanine/arginine aminopeptidase (upregulated also in adults), Acyl-CoA desaturase and serine carboxypeptidase. Aminopeptidase activities are detected in the midgut of mosquitoes in both larvae and adults [41]. The role of alanine aminopeptidase and more generally of aminopeptidase in thermal adaptation has been reported in different biological systems. In striped hamsters acclimated to cold (5 °C), alanine aminopeptidase activity was higher than in those exposed to hot temperatures (31 °C) [42], while it is well known that psychrophilic marine bacteria produce a cold-adapted aminopeptidase [43]. Concerning Acyl-CoA desaturase, there is much evidence on its involvement in cold adaptation processes: in the winged midge *Parochlus steinenii*, the extended acyl-CoA delta desaturase gene family underwent gene family expansion via multiple gene duplications for adaptation to the cold environments [44]; in *Drosophila* and Lepidoptera, the specific expression of some desaturases modulates cold adaptation mechanisms [45, 46]. Serine carboxypeptidases are expressed in mud crabs in response to cold exposition [47].

**Table 2:** Genes differentially expressed in larvae and adults reared at 4 °C and 15 °C, respectively

Gene symbol	Protein name	Log2FC larvae	*P*-adjusted larvae	Log2FC adults	*P*-adjusted adults
Shared genes in larvae and adults of *Aedes japonicus*					
*aapo1*	Alanine/arginine aminopeptidase	11.656	2.790e−07	10.181	4.440e−02
*abaq*	Quinolone resistance transporter	12.385	2.790e−08	13.691	8.400e−03
*aca13*	Putative calcium-transporting ATPase 13, plasma membrane-type	9.773	1.510e−04	11.425	4.970e−02
*acbp4*	Acyl-CoA-binding domain-containing protein 4	10.950	2.947e−06	9.561	4.890e−02
*acoB*	Acetoin:2,6-dichlorophenolindophenol oxidoreductase subunit beta	12.634	1.826e−09	12.130	4.190e−02
*acr*	Acrosin	12.487	2.510e−08	8.841	4.930e−02
*adhT*	Alcohol dehydrogenase	11.809	3.403e−08	11.759	4.620e−02
*aef1*	Adult enhancer factor 1	5.163	1.248e−02	10.375	3.780e−02
*aes*	Amino-terminal enhancer of split	12.574	2.158e−08	10.135	4.100e−02
*afg2*	ATPase family gene 2 protein	11.695	2.670e−06	11.511	4.870e−02
*aglA*	Probable alpha-glucosidase	12.723	4.090e−09	12.255	2.950e−02
*aldh9a1a*	Aldehyde dehydrogenase family	8.228	7.420e−03	12.342	4.090e−02
*aspC*	Aspartate aminotransferase	12.723	4.090e−09	10.232	3.480e−02
*baiA*	3alpha-hydroxysteroid dehydrogenase	10.742	1.159e−05	10.163	4.090e−02
*cyp72a613*	Cytochrome P450 CYP72A613	6.693	4.920e−03	11.863	4.510e−02
*cyp72a616*	Cytochrome P450 CYP72A616	12.842	8.839e−09	11.863	4.510e−02
*ksdD*	3-oxosteroid 1-dehydrogenase	9.216	1.220e−03	13.266	3.190e−02
*acr*	Acrosin	− 5.629	1.300e−03	− 10.732	3.229e−02
*aef1*	Adult enhancer factor 1	− 8.494	3.000e−04	− 9.639	4.840e−02
*ab*	Protein abrupt	− 12.959	1.129e−10	− 9.678	4.710e−02
Shared genes in larvae and adults of *Aedes koreicus*					
*aef1*	Adult enhancer factor 1	20.775	1.580e−04	20.580	3.287e−06

In *Ae. koreicus* adults, among many other genes likely involved in thermal regulation, we found upregulated genes encoding for cytochrome P450, fatty acyl-CoA reductase and mitochondrial adenine nucleotide transporter/translocase. Insect’s cytochrome P450 is well known to be involved in tolerance mechanisms [48, 49]; in some insects, such as the *Chrysomelidae, Galeruca daurica*, it is upregulated together with a fatty acyl-CoA reductase during recovery from cold stress [50]. Mitochondrial adenine nucleotide transporter/translocase has roles in thermal adaptation in several organisms as for example in thermal acclimation in the rainbow trout *Oncorhynchus mykiss* [51] or in response to thermal stress in *Apostichopus japonicus* [52].

Among the genes downregulated in larvae of *Ae. koreicus*, after exposure to 4 °C, we found a gene encoding a 5ʹ-AMP-activated protein kinase (catalytic subunit alpha-2); interestingly, activation of an AMP-activated protein kinase in response to temperature elevation has been reported in the zebra mussel, *Dreissena polymorpha* [53].

Two genes were found downregulated in adults of *Ae. koreicus* when exposed to cold conditions: an acetyl-CoA carboxylase (*acc*) and a steroid receptor seven-up, isoform A. *acc* is reported to have a role in thermal adaptation in sheep, more precisely its activity seems depressed in tissues exposed to cold [54]. The steroid receptor seven-up is involved in *Drosophila* oogenesis, thus influencing final egg output [55].

In *Ae. japonicus* larvae, among the many upregulated genes plausibly involved in the mechanisms of cold adaptation after exposure to 15 °C, there are some common to those previously described in *Ae. koreicus* (e.g. cytochrome P450). Among the many others of interest are those encoding some dehydrogenases (both alcohol and 6-phosphogluconate dehydrogenase), a multicopper oxidase, a glycosyl hydrolase and helicases. Indeed, in *Bactrocera dorsalis* low-temperature stress induced higher alcohol dehydrogenase activities in different life developmental stages with higher increase intensity in adults and pupae than in larvae [56]. 6-Phosphogluconate dehydrogenase is known to play a role in increasing cold tolerance in some plants [57, 58]. Multicopper oxidases are also involved in mechanisms of cold tolerance in different organisms such as plants and microbes [59, 60], while glycosyl hydrolases contribute to cold adaptation in many different organisms ranging from many plants to some yeasts up to the Antarctic springtail [61]. Some members of the helicase family behave similarly to different plants and algae [62, 63].

In adults of *Ae. japonicus*, among the many genes up-egulated after exposure to 15 °C, we should mention the members of the cytochrome P450 and Acyl-CoA desaturase families which play roles in cold adaptation have already been described [64]. Moreover, genes of the actin family, which in *Culex pipiens* are known to be expressed from early diapause to late diapause and in young non-diapaused adult mosquitoes reared at 18 °C [65], were found upregulated, as well as members of mitochondrial carriers and members of the methyltransferase superfamily. Interestingly, in some tick species, DNA methyltransferases are known to contribute to cold tolerance [66]. Other genes found upregulated are members of the aminotransferase family that, as shown in Corn Borer *Ostrinia nubilalis*, during diapause and cold hardening catalyze the production of l-alanine, an important cryoprotectant [67].

In both *Ae. japonicus* larvae and adults, the gene encoding the adult enhancing factor 1 (*aef1*) was found downregulated after exposure to 15 °C. Among other functions, this factor has been shown to bind the alcohol dehydrogenase adult enhancer site (AAE), thus regulating its transcription [68]. In adults, as already seen in *Ae. koreicus*, a gene encoding a 5ʹ-AMP-activated protein kinase catalytic sub-unit alpha was also downregulated when exposed to cold.

## Discussion

Mosquito invasive species, having the potential to transmit a range of different pathogens, pose significant public health problems where they establish, and their ranges and potential impacts are shifting with climate change. This is well demonstrated by the 30-year experience on the stabilization of *Ae. albopictus* in Europe and, more recently, on the impact of the transmission of urban malaria in relation to the arrival of *An. stephensi* in Africa [2]. Although some information about genome organization has been very partially provided for *Ae. koreicus* [9], a more detailed knowledge of the basic biology of the new European invasive species *Ae. koreicus* and *Ae. japonicus* is a fundamental prerequisite to control these insect vectors. Hence, genome sequencing of both species may provide insight into the genetic basis of their competence for pathogen transmission and for the development of species-specific control methods.

Both species are characterized by a genome size and GC content comparable to other aedines genomes (*Ae. albopictus* and *Ae. aegypti*).

Comparing the metrics of our assembly to the previous draft genome [9], we highlighted an improvement in the Quast scores as in the genome length (1.24 Gbp vs 879 Mbp) and in the N50 (190,716 vs 18,000) as well as in the BUSCO score of the *Ae. koreicus* assembly (91.8% vs. 74.52%) of genes present in the Diptera Database.

Furthermore, the two species are phylogenetically correlated regarding all the other mosquito species considered. This suggests common mechanisms of adaptation to eco-ethological contexts and could explain their almost contemporary appearance in large areas of Mediterranean and central Europe.

Consequently, forecasting changes in the expansion of the regions that are suitable for invasion of *Aedes* vectors and *Aedes*-borne viruses, regarding dengue, chikungunya and zika, is a key element of public health preparedness. Moreover, the insecticide resistance developed by several mosquito vectors is undermining the effectiveness of their control. Thus, we focused on those genes implicated in both thermal adaptation and insecticide resistance. The analysis of both group of genes revealed some intriguing features. Both *Ae. koreicus* and *Ae. japonicus* are characterized by species-specific sets of genes as well as genes that are shared between these two species but not by other aedines. Considering the function(s) of the proteins encoded by these genes (e.g. decarboxylases), it is very likely that most, if not all, of these genes drive the specific behaviour of the two mosquito species in climatic adaptation and insecticide resistance. This is further substantiated by the RNA-seq analysis following exposure of larvae and adults to different temperatures. Indeed, as reported in the results, the expression of several genes has shown to be strongly modulated by the temperature and some of these genes seem to be involved in the adaptation to low temperatures and could, consequently, contribute on the one hand to a better understanding of the mechanisms underlying the geographical distributions of the two invasive species, on the other to better monitor and control the dispersion of the two species. The ability to monitor and control vector mosquitoes is also supported by the ability to use insecticides and biocides wisely in relation to the possible onset of insecticide-resistance. In this frame, the identification of genes plausibly involved in possible insecticide resistance mechanisms (e.g. chitin synthases) can represent an excellent basis to establish further monitoring and control programmes.

## Conclusions

Despite the need for future corroborating studies, the sequencing of the two genomes and the analysis of the two selected groups of genes pave the way to the possibility of specific control strategies aimed at limiting the risks associated with the recent introduction of the two invasive species in Europe.

### Supplementary Information

**Additional file 1: Table S1.** Total raw reads count.

**Additional file 2: Table S2.** Thermal adaptation genes in *Aedes koreicus* and *Ae. japonicus*.

**Additional file 3: Table S3.** GO Enrichment analysis of thermal adaptation genes.

**Additional file 4: Table S4.** Insecticide resistance genes found in *Aedes koreicus* and *Ae. japonicus*.

**Additional file 5: Table S5.** Differentially expressed transcripts.

**Additional file 6: Table S6.** Gene Ontology (GO) enrichment analysis of genes differentially expressed in *Aedes koreicus* and *Ae. japonicus*.

## Data Availability

All the data that support the genome assembly and RNA analysis of *Ae. koreicus* and *Ae. japonicus* have been deposited in the NCBI and can be accessed with BioProject accession numbers PRJNA947548 (https://dataview.ncbi.nlm.nih.gov/object/PRJNA947548?reviewer=vluhmg7o407263tdhui37m0u56) and PRJNA947978 (https://dataview.ncbi.nlm.nih.gov/object/PRJNA947978?reviewer=jsp2muuuu89t76qt9tds28kn1b), respectively.

## Abbreviations

Ajap1: De novo genome assembly of *Aedes japonicus*
Akor1: De novo genome assembly of the *Aedes koreicus*
ABC: ATP-binding cassette
ESTs: Expressed sequence tags
RNA-seq: RNA sequencing
PE: Paired-end reads
BUSCO: Benchmarking Universal Single-Copy Orthologs
KEGGS: Kyoto Encyclopedia of Genes and Genomes
iTOL: Interactive Tree Of Life
STRIDE: Species Tree Root Inference from gene Duplication Events
STAG: Species Tree Inference from All Genes
LoRDEC: Long Read DBG Error Correction
SPAdes: St. Petersburg genome assembler
QUAST: QUality ASsessment Tool
MCRA: Most common recent ancestor
AMP: Adenosine monophosphate
ATP: Adenosine triphosphate
NAD(P)H: Nicotinamide adenine dinucleotide phosphate
P450: P450 monooxygenases
BTB: Broad-complex, tramtrack and bric-a-brac
CCEs: Carboxyl/cholinesterases
GSTs: Glutathione-*S*-transferases
UGTs: UDP-glucuronosyltransferase
ABC: ATP-binding cassette
acc: Acetyl-CoA carboxylase
aef1: Adult enhancer factor 1
AAE: Alcohol dehydrogenase adult enhancer site
GO: Gene Ontology
CoA: Coenzyme A

## References

[1] Schaffner F, Medlock JM, Van Bortel W (2013). Public health significance of invasive mosquitoes in Europe. Clin Microbiol Infect..

[2] Vogel G (2022). Invasive mosquito adds to Africa's malaria toll. Science.

[3] Faulde MK, Rueda LM, Khaireh BA (2014). First record of the Asian malaria vector *Anopheles stephensi* and its possible role in the resurgence of malaria in Djibouti. Horn of Africa Acta Trop.

[4] https://www.science.org/content/article/spread-city-loving-malaria-mosquitoescould-pose-grave-threat-africa. 10.1126/science.abe8052

[5] Schaffner F, Chouin S, Guilloteau J (2003). First record of *Ochlerotatus* (Finlaya) *japonicus japonicus* (Theobald, 1901) in metropolitan France. J Am Mosq Control Assoc.

[6] Versteirt V, De Clercq EM, Fonseca DM, Pecor J, Schaffner F, Coosemans M (2012). Bionomics of the established exotic mosquito species *Aedes koreicus* in Belgium, Europe. J Med Entomol.

[7] Cebrián-Camisón S, Martínez-de la Puente J, Figuerola J (2020). A literature review of host feeding patterns of invasive. Insects.

[8] Müller P, Engeler L, Vavassori L, Suter T, Guidi V, Gschwind M (2020). Surveillance of invasive *Aedes* mosquitoes along Swiss traffic axes reveals different dispersal modes for *Aedes albopictus* and *Ae. japonicus*. PLoS Negl Trop Dis.

[9] Kurucz K, Zeghbib S, Arnoldi D, Marini G, Manica M, Michelutti A (2022). *Aedes koreicus*, a vector on the rise: pan-European genetic patterns, mitochondrial and draft genome sequencing. PLoS ONE.

[10] Montarsi F, Martini S, Dal Pont M, Delai N, Ferro Milone N, Mazzucato M (2013). Distribution and habitat characterization of the recently introduced invasive mosquito *Aedes koreicus* [*Hulecoeteomyia koreica*], a new potential vector and pest in north-eastern Italy. Parasit Vectors.

[11] Li H (2015). BFC: correcting Illumina sequencing errors. Bioinformatics.

[12] Salmela L, Rivals E (2014). LoRDEC: accurate and efficient long read error correction. Bioinformatics.

[13] Kolmogorov M, Yuan J, Lin Y, Pevzner PA (2019). Assembly of long, error-prone reads using repeat graphs. Nat Biotechnol.

[14] Kundu R, Casey J, Sung WK. HyPo: Super fast & accurate polisher for long read genome assemblies; 2019. 10.1101/2019.12.19.882506

[15] Warren RL, Yang C, Vandervalk BP, Behsaz B, Lagman A, Jones SJ (2015). LINKS: scalable, alignment-free scaffolding of draft genomes with long reads. Gigascience.

[16] Coombe L, Li JX, Lo T, Wong J, Nikolic V, Warren RL (2021). LongStitch: high-quality genome assembly correction and scaffolding using long reads. BMC Bioinform.

[17] Guan D, McCarthy SA, Wood J, Howe K, Wang Y, Durbin R (2020). Identifying and removing haplotypic duplication in primary genome assemblies. Bioinformatics.

[18] Mikheenko A, Prjibelski A, Saveliev V, Antipov D, Gurevich A (2018). Versatile genome assembly evaluation with QUAST-LG. Bioinformatics.

[19] Manni M, Berkeley MR, Seppey M, Zdobnov EM (2021). BUSCO: assessing genomic data quality and beyond. Curr Protoc.

[20] Bolger AM, Lohse M, Usadel B (2014). Trimmomatic: a flexible trimmer for Illumina sequence data. Bioinformatics.

[21] Andrews S. FastQC: a quality control tool for high throughput sequence data. Babraham Bioinformatics; 2010. Available online at: http://www.bioinformatics.babraham.ac.uk/projects/fastqc

[22] Bushmanova E, Antipov D, Lapidus A, Prjibelski AD (2019). rnaSPAdes: a de novo transcriptome assembler and its application to RNA-Seq data. Gigascience.

[23] Patro R, Duggal G, Love MI, Irizarry RA, Kingsford C (2017). Salmon provides fast and bias-aware quantification of transcript expression. Nat Methods.

[24] Love MI, Huber W, Anders S (2014). Moderated estimation of fold change and dispersion for RNA-seq data with DESeq2. Genome Biol.

[25] Bryant DM, Johnson K, DiTommaso T, Tickle T, Couger MB, Payzin-Dogru D (2017). A tissue-mapped Axolotl De novo transcriptome enables identification of limb regeneration factors. Cell Rep.

[26] Consortium U (2023). UniProt: the Universal Protein Knowledgebase in 2023. Nucleic Acids Res.

[27] Chen EY, Tan CM, Kou Y, Duan Q, Wang Z, Meirelles GV (2013). Enrichr: interactive and collaborative HTML5 gene list enrichment analysis tool. BMC Bioinform.

[28] Holt C, Yandell M (2011). MAKER2: an annotation pipeline and genome-database management tool for second-generation genome projects. BMC Bioinform.

[29] Korf I (2004). Gene finding in novel genomes. BMC Bioinform.

[30] Stanke M, Schöffmann O, Morgenstern B, Waack S (2006). Gene prediction in eukaryotes with a generalized hidden Markov model that uses hints from external sources. BMC Bioinform.

[31] Tarailo-Graovac M, Chen N (2009). Using RepeatMasker to identify repetitive elements in genomic sequences. Curr Protoc Bioinform.

[32] Jones P, Binns D, Chang HY, Fraser M, Li W, McAnulla C (2014). InterProScan 5: genome-scale protein function classification. Bioinformatics.

[33] Emms DM, Kelly S (2015). OrthoFinder: solving fundamental biases in whole genome comparisons dramatically improves orthogroup inference accuracy. Genome Biol.

[34] Letunic I, Bork P (2021). Interactive Tree Of Life (iTOL) v5: an online tool for phylogenetic tree display and annotation. Nucleic Acids Res.

[35] Herrmann M, Yampolsky LY (2021). False and true positives in arthropod thermal adaptation candidate gene lists. Genetica.

[36] Gertz EM, Yu YK, Agarwala R, Schäffer AA, Altschul SF (2006). Composition-based statistics and translated nucleotide searches: improving the TBLASTN module of BLAST. BMC Biol.

[37] Faucon F, Dusfour I, Gaude T, Navratil V, Boyer F, Chandre F (2015). Identifying genomic changes associated with insecticide resistance in the dengue mosquito *Aedes aegypti* by deep targeted sequencing. Genome Res.

[38] Matthews BJ, Dudchenko O, Kingan SB, Koren S, Antoshechkin I, Crawford JE (2018). Improved reference genome of *Aedes aegypti* informs arbovirus vector control. Nature.

[39] Palatini U, Masri RA, Cosme LV, Koren S, Thibaud-Nissen F, Biedler JK (2020). Improved reference genome of the arboviral vector *Aedes albopictus*. Genome Biol.

[40] Main BJ, Marcantonio M, Johnston JS, Rasgon JL, Brown CT, Barker CM (2021). Whole-genome assembly of *Culex tarsalis*. G3 (Bethesda).

[41] Zhao ZJ, Chi QS, Liu QS, Zheng WH, Liu JS, Wang DH. The shift of thermoneutral zone in striped hamster acclimated to different temperatures. PLoS One. 2014;6;9(1):e84396. 10.1371/journal.pone.0084396. **PMID: 24400087; PMCID: PMC3882234**. 10.1371/journal.pone.0084396 PMC3882234 24400087

[42] Zhao ZJ, Chi QS, Liu QS, Zheng WH, Liu JS, Wang DH (2014). The shift of thermoneutral zone in striped hamster acclimated to different temperatures. PLoS ONE.

[43] Huston AL, Haeggström JZ, Feller G (2008). Cold adaptation of enzymes: structural, kinetic and microcalorimetric characterizations of an aminopeptidase from the Arctic psychrophile *Colwellia psychrerythraea* and of human leukotriene A(4) hydrolase. Biochim Biophys Acta.

[44] Kim H, Kim HW, Lee JH, Park J, Lee H, Kim S (2022). Gene family expansions in Antarctic winged midge as a strategy for adaptation to cold environments. Sci Rep.

[45] Suito T, Nagao K, Takeuchi K, Juni N, Hara Y, Umeda M (2020). Functional expression of Δ12 fatty acid desaturase modulates thermoregulatory behaviour in *Drosophila*. Sci Rep.

[46] Min Q, Cheng S, Xi J, Xin T, Xia B, Zou Z (2017). Differential expression patterns of two delta-9-acyl-CoA desaturases in. Ecol Evol.

[47] Chen J, Leng T, Jiang YM, Chen XB, Liu ZM (2022). RNA-seq analysis of the differential response to low-temperature stress in two morphs of mud crabs (*Scylla paramamosain*). Comp Biochem Physiol Part D Genomics Proteomics.

[48] Franke K, Karl I, Centeno TP, Feldmeyer B, Lassek C, Oostra V (2019). Effects of adult temperature on gene expression in a butterfly: identifying pathways associated with thermal acclimation. BMC Evol Biol.

[49] Huang HJ, Xue J, Zhuo JC, Cheng RL, Xu HJ, Zhang CX (2017). Comparative analysis of the transcriptional responses to low and high temperatures in three rice planthopper species. Mol Ecol.

[50] Zhou XR, Shan YM, Tan Y, Zhang ZR, Pang BP (2019). Comparative analysis of transcriptome responses to cold stress in *Galeruca daurica* (Coleoptera: Chrysomelidae). J Insect Sci.

[51] Kraffe E, Marty Y, Guderley H (2007). Changes in mitochondrial oxidative capacities during thermal acclimation of rainbow trout *Oncorhynchus mykiss*: roles of membrane proteins, phospholipids and their fatty acid compositions. J Exp Biol.

[52] Liu QN, Chai XY, Tu J, Xin ZZ, Li CF, Jiang SH (2016). An adenine nucleotide translocase (ANT) gene from *Apostichopus japonicus*; molecular cloning and expression analysis in response to lipopolysaccharide (LPS) challenge and thermal stress. Fish Shellfish Immunol.

[53] Jost JA, Keshwani SS, Abou-Hanna JJ (2015). Activation of AMP-activated protein kinase in response to temperature elevation shows seasonal variation in the zebra mussel, *Dreissena polymorpha*. Comp Biochem Physiol A Mol Integr Physiol.

[54] Moibi JA, Ekpe ED, Christopherson RJ (2000). Acetyl-CoA carboxylase and fatty acid synthase activity and immunodetectable protein in adipose tissues of ruminants: effect of temperature and feeding level. J Anim Sci.

[55] Weaver LN, Drummond-Barbosa D (2019). The nuclear receptor seven up functions in adipocytes and oenocytes to control distinct steps of *Drosophila* oogenesis. Dev Biol.

[56] Wang J, Zeng L, Han Z (2014). An assessment of cold hardiness and biochemical adaptations for cold tolerance among different geographic populations of the *Bactrocera dorsalis* (Diptera: Tephritidae) in China. J Insect Sci.

[57] Tian Y, Peng K, Bao Y, Zhang D, Meng J, Wang D (2021). Glucose-6-phosphate dehydrogenase and 6-phosphogluconate dehydrogenase genes of winter wheat enhance the cold tolerance of transgenic *Arabidopsis*. Plant Physiol Biochem.

[58] Sardesai N, Babu CR (2001). Poly-beta-hydroxybutyrate metabolism is affected by changes in respiratory enzymatic activities due to cold stress in two psychrotrophic strains of *Rhizobium*. Curr Microbiol.

[59] Xu X, Zhang Y, Liang M, Kong W, Liu J (2022). The citrus laccase gene CsLAC18 contributes to cold tolerance. Int J Mol Sci.

[60] Karmacharya J, Shrestha P, Han SR, Park H, Oh TJ (2022). Complete genome sequencing of polar *Arthrobacter* sp. PAMC25284, copper tolerance potential unraveled with genomic analysis. Int J Microbiol.

[61] Song JM, Hong SK, An YJ, Kang MH, Hong KH, Lee YH (2017). Genetic and structural characterization of a thermo-tolerant, cold-active, and acidic endo-β-1,4-glucanase from antarctic springtail, *Cryptopygus antarcticus*. J Agric Food Chem.

[62] Peng Z, Liu G, Huang K (2021). Cold adaptation mechanisms of a snow alga *Chlamydomonas nivalis* during temperature fluctuations. Front Microbiol.

[63] Wang Y, Liu X, Gao H, Zhang HM, Guo AY, Xu J (2020). Early stage adaptation of a mesophilic green alga to antarctica: systematic increases in abundance of enzymes and LEA proteins. Mol Biol Evol.

[64] Iqbal T, Chakraborty S, Murugan S, Das D (2022). Metalloenzymes for fatty acid-derived hydrocarbon biosynthesis: nature's cryptic catalysts. Chem Asian J.

[65] Kim M, Robich RM, Rinehart JP, Denlinger DL (2006). Upregulation of two actin genes and redistribution of actin during diapause and cold stress in the northern house mosquito, *Culex pipiens*. J Insect Physiol.

[66] Agwunobi DO, Zhang M, Shi X, Zhang S, Wang T, Masoudi A (2021). DNA methyltransferases contribute to cold tolerance in ticks. Front Vet Sci.

[67] Uzelac I, Avramov M, Čelić T, Vukašinović E, Gošić-Dondo S, Purać J (2020). Effect of cold acclimation on selected metabolic enzymes during diapause in the European corn borer *Ostrinia nubilalis* (Hbn). Sci Rep.

[68] Potter JJ, Mezey E, Yang VW (1994). The adult enhancer factor-1, a *Drosophila melanogaster* transcriptional repressor, modulates the promoter activity of the rat class-I alcohol dehydrogenase-encoding gene. Gene.

