# Supplementary material for: De novo genome assembly of the invasive mosquito species Aedes japonicus and Aedes koreicus
Source: Parasit Vectors. 2023 Nov 20;16:427. doi: 10.1186/s13071-023-06048-w (PMC10658958; doi:10.1186/s13071-023-06048-w)
Supplement: Supplementary file 6 — Additional file 6: Table S6. Gene Ontology (GO) enrichment analysis of genes differentially expressed in Aedes koreicus and Ae. japonicus. [file 13071_2023_6048_MOESM6_ESM.doc]

**Additional file 6: Table S6. Gene Ontology (GO) enrichment analysis of genes differentially expressed in *Aedes koreicus* and *Aedes japonicus.*** Significant GO terms categories representing both Biological Process and Molecular Function of differentially expressed genes (p-adjusted < 0.05) in *Ae. koreicus* larvae (**A)** in *Ae. koreicus* adults (**B)** in *Ae. japonicus* adults (**C**). No significant terms were enriched for *Ae. japonicus* larvae. For each significant GO categories, the p-value, p-adjusted, odds ratio, and combined score were accordingly included.

**A**

| Name | p-value | Adjusted  p-value | Odds  Ratio | Combined  score | Category |
| --- | --- | --- | --- | --- | --- |
| ATP-activated inward rectifier potassium channel activity (GO:0015272) | 0.002997 | 0.04453 | 444.11 | 2580.41 | Molecular Function |
| inward rectifier potassium channel activity  (GO:0005242) | 0.01243 | 0.04453 | 92.44 | 405.55 | Molecular Function |
| actin monomer binding  (GO:0003785) | 0.01243 | 0.04453 | 92.44 | 405.55 | Molecular Function |
| potassium ion transmembrane transporter activity  (GO:0015079) | 0.01589 | 0.04453 | 71.54 | 296.32 | Molecular Function |
| non-membrane spanning protein tyrosine kinase activity (GO:0004715) | 0.01737 | 0.04453 | 65.22 | 264.33 | Molecular Function |
| phosphotyrosine residue binding  (GO:0001784) | 0.01835 | 0.04453 | 61.59 | 246.23 | Molecular Function |
| protein phosphorylated amino acid binding  (GO:0045309) | 0.02081 | 0.04453 | 54.06 | 209.35 | Molecular Function |
| manganese ion binding  (GO:0030145) | 0.02375 | 0.04453 | 47.15 | 176.34 | Molecular Function |
| cation-transporting ATPase complex  (GO:0090533) | 0.007973 | 0.03986 | 147.96 | 714.91 | Cellular Component |

**B**

| Name | p-value | Adjusted  p-value | Odds  Ratio | Combined score | Category |
| --- | --- | --- | --- | --- | --- |
| fucose binding  (GO:0042806) | 0.005488 | 0.04060 | 237.79 | 1237.70 | Molecular Function |
| CoA carboxylase activity  GO:0016421) | 0.006583 | 0.04060 | 190.22 | 955.53 | Molecular Function |

**C**

| Name | p-value | Adjusted  p-value | Odds Ratio | Combined score | Category |
| --- | --- | --- | --- | --- | --- |
| purine nucleoside bisphosphate metabolic process (GO:0034032) | 1.362e-7 | 0.00004289 | 22.20 | 350.91 | Biological Process |
| ribonucleoside bisphosphate metabolic process (GO:0033875) | 1.362e-7 | 0.00004289 | 22.20 | 350.91 | Biological Process |
| acyl-CoA metabolic process (GO:0006637) | 8.603e-6 | 0.001807 | 14.67 | 171.07 | Biological Process |
| acyl-CoA dehydrogenase activity  (GO:0003995) | 1.842e-4 | 0.01934 | 37.51 | 322.54 | Molecular Function |
